# Methane reduction by quercetin, tannic and salicylic acids: influence of molecular structures on methane formation and fermentation in vitro

**DOI:** 10.1038/s41598-023-43041-w

**Published:** 2023-09-25

**Authors:** Natalja P. Nørskov, Marco Battelli, Mihai V. Curtasu, Dana W. Olijhoek, Élisabeth Chassé, Mette Olaf Nielsen

**Affiliations:** 1 Department of Animal and Veterinary Sciences, Aarhus University, Blichers Allé 20, 8830 Tjele, Denmark; 2 Department of Agricultural and Environmental Sciences-Production, Landscape, Agroenergy, Università degli Studi di Milano, Via Celoria 2, 20133 Milan, Italy

**Keywords:** Climate-change mitigation, Environmental biotechnology

## Abstract

Plant secondary metabolites (PSMs) can potentially reduce ruminal methane formation. However, related to differences in their molecular structures, it is not yet clear what causes an anti-methanogenic effect. In an in vitro system simulating rumen fermentation, we investigated the impact of eight compounds with distinct chemical characteristics (gallic and salicylic acids, tannic acid, catechin, epicatechin, quercetin, rutin, and salicin) when added to a basal feed (maize silage) at a concentration of 12% of the feed dry matter. After 48 h of incubation in buffered rumen fluid, methane production was significantly lowered by quercetin (43%), tannic acid (39%) and salicylic acid (34%) compared to the control (maize silage alone) and without changes in total volatile fatty acid production during fermentation. No other PSM reduced methane formation as compared to control but induced significant differences on total volatile fatty acid production. The observed differences were related to lipophilicity, the presence of double bond and carbonyl group, sugar moieties, and polymerization of the compounds. Our results indicate the importance of distinct molecular structures of PSMs and chemical characteristics for methane lowering properties and volatile fatty acid formation. Further systematic screening studies to establish the structure–function relationship between PSMs and methane reduction are warranted.

## Introduction

Climate change is associated with emission of greenhouse gasses (GHG) such as carbon dioxide and methane. Even though methane is emitted at lower levels compared to carbon dioxide, it is a more potent GHG with a 100-year global warming potential 28 times higher than that of carbon dioxide^1^. Ruminants can obtain energy and nutrients from fibrous feeds due to the fermentation processes carried out by microorganisms living in the rumen in a symbiotic relation with the ruminant animal. During fermentation, microorganisms release volatile fatty acids (VFAs), which represent an important energy source for ruminants. However, methane is also produced during feed fermentation in the rumen by rumen methanogens from carbon dioxide and hydrogen^2^. Dietary strategies to interfere with enteric methane formation has been the subject of intense studies in the last decade, and this has included the use of different plant biomasses or extracts rich in plant secondary metabolites (PSMs) as methane mitigation strategy^3–5^.

PSMs are natural compounds produced by plants as part of their defence mechanism, pigmentation, growth, reproduction, and many other functions^6^. Plants produce a plethora of different molecular structures, some of which have been claimed to have the potential to inhibit enteric methane from ruminants^7^. Decades of research have shown that some particular tannins, flavonoids and phenolic acids, generally also known as phenolic compounds (PCs), have the potential to modulate ruminal fermentation^8–11^. However, research to fill the knowledge gap with respect to structure–activity relationships of these compounds on methane formation is still in its infancy.

**Figure 1:**
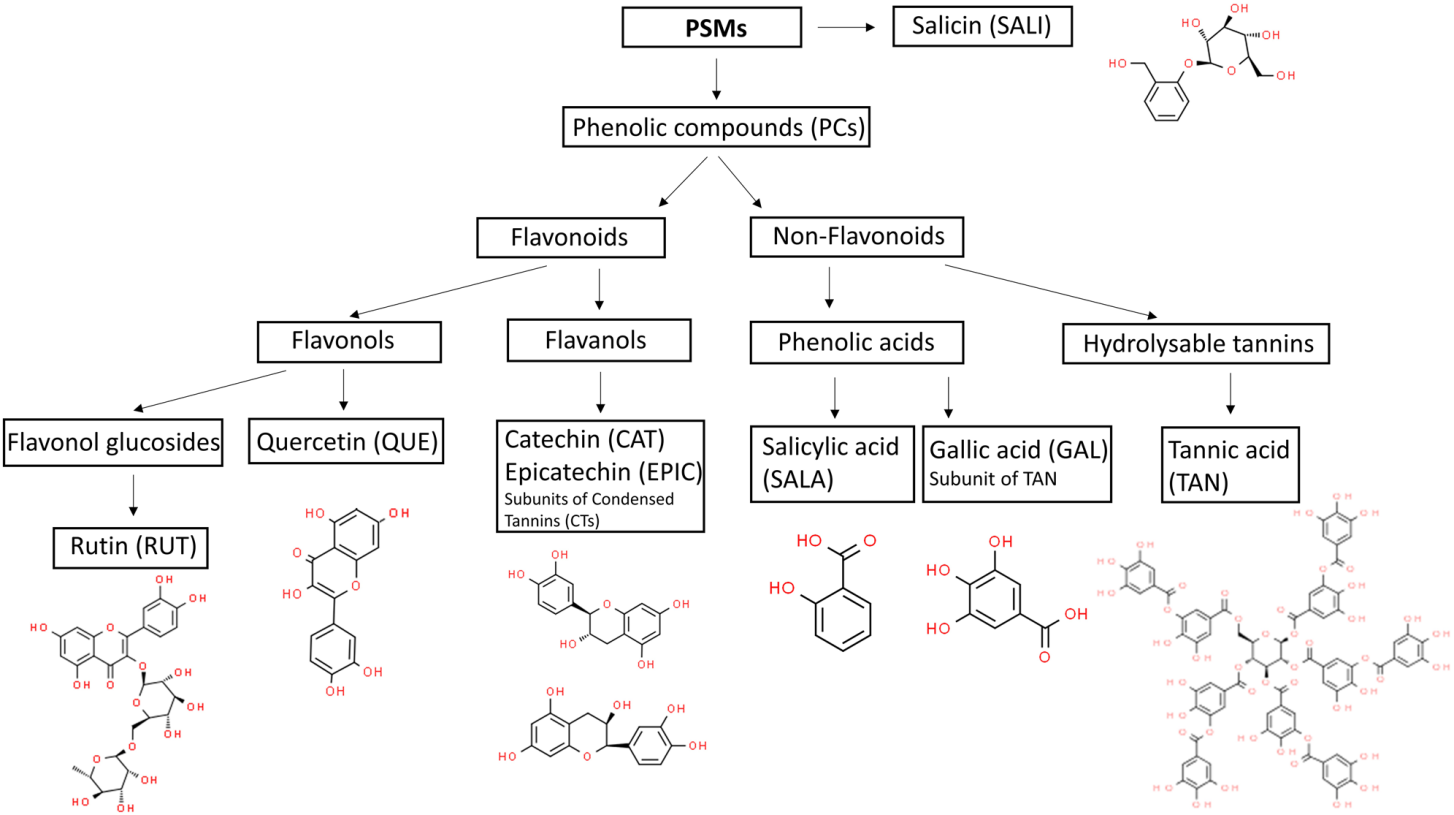
Classification and molecular structures of Plant Secondary Metabolites (PSMs) used in this study. The chemical structures were downloaded from ChemSpider database www.chemspider.com/Chemical-Structure (accessed 14:30, Aug 28, 2023): Rutin, CSID: 4444362; Quercetin, CSID: 4444051; Catechin, CSID: 8711; Epicatechin, CSID: 65230; Salicylic acid, CSID: 331; Gallic acid, CSID: 361; Tannic acid, CSID: 17286569; Salicin, CSID: 388601.

Tannins with methane mitigating potential are commonly classified into two classes, hydrolysable tannins and condensed tannins^12^. Hydrolysable tannins are a group of compounds, classified as non-flavonoids that contain a polyol core (commonly glucose) at the centre of the molecule, whose hydroxyl groups are partially or completely esterified with the carboxyl groups of gallic acid (GAL). GAL is thereby a subunit of tannic acid (TAN), Fig. 1. This molecular structure constitutes the simplest hydrolysable tannin, tannic acid (TAN) also known as gallotannin (Fig. 1)^9, 13–15^. TAN contains no carboxyl group in contrast to GAL, but the TAN molecule is nevertheless slightly acidic because of multiple phenolic hydroxyl groups^16^. The subunit GAL is by itself classified as a simple phenolic acid and is more acidic than TAN because of its carboxyl group. GAL contains three hydroxyl groups and is therefore relatively soluble in water^16^. Both hydrolysable tannins and condensed tannins are polymers, large molecules with molecular weight > 500 Da as compared to their monomeric subunits. Condensed tannins belong to the broad class of flavonoids, with highly varying molecular weights (MWs) depending on the degree of polymerization. Condensed tannins are oligomers of flavanol subunits^12, 17^. There are several types of flavanol subunits found in condensed tannins. The most common ones are catechin (CAT) and epicatechin (EPIC)^12^ (Fig. 1). Despite the high number of hydroxyl groups (5 OH), CAT and its isomeric structure, EPIC, have low solubility in water^16^. Besides tannins, other flavonoids have also been mentioned in the literature in association with reduction of enteric methane emission^9^. Flavonoids such as quercetin (QUE) and rutin (RUT) are PCs belonging to the class of flavonols. RUT is a glucoside of QUE (quercetin-3-*O*-rutinoside)^13^ (Fig. 1). Flavonols are poorly soluble in water^16^. Plants accumulate flavonols like RUT as aglycons linked to a variable number of different sugar moieties by β-glycosidic bonds (Fig. 1), mainly at position 3 of the C ring^18^. The conjugation with the sugar moiety may reduce their bioactivity; however, it improves their solubility in water and, thereby, their bioavailability^18^. Generally, flavonoids are composed of two phenyl rings (A and B rings) and a heterolytic C ring with carbon structure C_6_–C_3_–C_6_^19, 20^. The structural difference between flavanols and flavonols is characterised by flavonols having a double bond in the C ring between C-2 and C-3 in conjunction with the 4-carbonyl group, but a similar number and position of hydroxyl groups^20^. The absence of double bonds in the C ring between C-2 and C-3 of flavanols determine their stereoisomeric properties^19, 20^.

Both in vitro simulation of rumen fermentation and in vivo studies with cows, goats, and sheeps, have been performed with feed incubated in sacco in the rumen or with feeding of biomass from plants rich in PSMs and extracts of varied origins as reviewed in different works^11, 21–23^. For example, a large in vitro screening study with 450 plant species showed methane-reducing properties of 35 plant species. Six of the species reduced the in vitro production of methane per gram of dry matter (DM) of standard feeds by more than 25% compared to control incubations of the standard feed alone, and without detrimental effects on feed degradability, total gas production (TGP) or VFA production^24^. Bodas et al.^24^ concluded that certain plants and plant extracts have the potential to inhibit methane emission in vitro. However, details on the composition and concentration of PSMs in the plant biomass or extracts used in the majority of the published studies have been missing. Therefore, the specific PSMs responsible for any anti-methanogenic effect were not properly identified. Another approach to study the bioactivity of a particular PSM or PSM class is to screen PSMs as pure chemical standards. To our knowledge only two in vitro studies have been performed with multiple PCs as pure compounds to assess their ability to modify rumen fermentations and reduce methane emission^18, 25^. To obtain in-depth knowledge on the mode of action of single PSM and to further bridge the gap of knowledge on the influence of different molecular structures of PSMs and their corresponding chemical characteristics on methane formation, studies with pure compounds are warranted.

We hypothesized that pure PSMs have anti-methanogenic potential as well as distinct actions on rumen fermentation patterns depending on their polymeric or monomeric structures, isomerization, number of phenol and hydroxyl groups, the presence of carboxyl group and conjugation with sugar moiety. To test this hypothesis, a selection of pure PSM compounds shown in Fig. 1, which differed in the above-mentioned molecular structures, were dissolved and incubated with a feed (maize silage) in an in vitro system simulating rumen fermentation to evaluate the impact on methane formation and VFA production during 48 h of incubation. Besides the PSMs which were mentioned in the introduction, we have investigated the anti-methanogenic effect of salicylic acid (SALA) and salicin (SALI) (Fig. 1). These compounds have been included in the study based on the consideration that they are found in high concentrations in *Salix* spp., a plant biomass that has been reported to possess anti-methanogenic potential^24, 26^. To our knowledge this is the first study with SALA and SALI.

## Materials and methods

### Chemicals

The pure compounds: phenolic acids; SALA (CAS number: 69-72-7; purity: ≥ 99%, Molecular Weight (MW) 138.12 Da) and GAL (CAS number: 149-91-7; purity: ≥ 98.5%, MW 170.12 Da); hydrolysable tannin, TAN (CAS number: 1401-55-4; purity: ACS reagent, MW 1701,20 Da), flavanols; CAT (CAS number: 225937-10-0; purity: ≥ 96%, MW 290.27 Da) and EPIC (CAS number: 490-46-0; purity: ≥ 90%, MW 290.27 Da), flavonols; QUE (CAS number: 117-39-5, purity: ≥ 95%, MW 302.24 Da) and RUT (CAS number: 207671-50-9; purity: ≥ 94%, MW 610.52 Da), and non-PC SALI (CAS number: 138-52-3; purity: ≥ 99%, MW 286.28 Da) were purchased as dry powders from Sigma-Aldrich (Merck KGaA, Darmstadt, Germany).

Each compound (0.060 g) was dissolved in 2 mL of either dimethyl sulfoxide (DMSO; Sigma) or pure water to reach the concentration of 30 mg/mL. Due to poor solubility of CAT, EPIC, QUE, RUT, GAL, and SALA in water, these compounds were dissolved in DMSO, while pure water was used to dissolve SALI and TAN, Figure S1.

### In vitro simulation of rumen fermentation

The impact of the PSMs on rumen fermentation characteristics was assessed in vitro by incubating maize silage (MS) as a standard feed in buffered rumen inoculum under anaerobic conditions, with or without the addition of a PSM. The experiment was conducted at AU Viborg (Tjele, Denmark). The procedures involving rumen cannulation of cows and sampling of rumen fluid from these cows were approved by the Animal Experiments Inspectorate in accordance with the guidelines established by directive 2010/63/EU and current Danish legislation (law no. 474, May 14, 2014), and were in compliance with ARRIVE guidelines. On the morning of each experiment, rumen fluid was collected half an hour before morning feeding from three rumen-cannulated non-gestating dry Holstein cows housed at the experimental facility at Aarhus University, Foulum, Denmark. The handling and care of the cows complied with the guidelines set out by the Danish Ministry of Environment and Food (2020) (Act No. 2028, 2020) with respect to animal experimentation and care of animals under study. The cows were fed at maintenance level with a standard diet composed of straw, hay, and a concentrate mixture^27^. The rumen fluid was immediately transferred to preheated thermo bottles and transported to the laboratory within 30 min after sampling, where it was filtered through two layers of moist cheesecloth, and the pH of the filtrate was measured. The final in vitro inoculum consisted of filtered rumen fluid and a buffer solution (redox indicator, reducing agent, buffer, and macro- and micromineral solutions as described by Menke and Steingass^28^) mixed in a 2:1 ratio. During the preparation of the buffer solution and final inoculum, the solution was continuously flushed with N_2_ to maintain anaerobic conditions.

Incubations were conducted in Duran^®^ bottles (capacity: 132 ± 1.1 mL) containing 0.5 g of MS, and 90 mL of buffered rumen fluid with or without 2 mL solution of each PSM, to reach the concentration of 12% (w/w) of each PSM on feed DM basis. The high inclusion rate was chosen to ensure that any effects on methane formation by the individual PSMs were detectable. The MS was freeze-dried and milled through a 2-mm sieve on a centrifugal mill (Ultra Centrifugal Mill ZM 200, Verder Scientific, Hann, Germany). To account for possible effects of DMSO on fermentation, negative controls were included with bottles containing 2 mL of pure MilliQ water (MS) or 2 mL of pure DMSO (MS-DMSO) without PSM. The headspace of the bottles was flushed with N_2_ before the ANKOM pressure sensor module (AnkomTechnology, Macedon, NY, USA) was fitted on top of the bottle (Supplementary Fig. S2). All the bottles were incubated in an incubator shaker (New BrunswickTM Excella^®^ E25R, Eppendorf, Hamburg, Germany) at 38.5 °C and 50 rpm oscillation for 48 h. All the treatments were tested in triplicate in each of two separate incubation runs. In each run, 3 blanks (bottles containing only buffered rumen fluid without MS) were also included.

During the incubations, the pressure changes in the headspace of the bottles were continuously recorded every 10 min as a difference with respect to the atmospheric pressure. The produced gas was released from the headspace through the opening of a valve for 250 ms whenever the pressure inside the bottle reached 0.75 psi above ambient pressure, and the accumulated gas production was automatically calculated. The released gas was collected in a gas-tight 1 L Aluminium Bag CEK-1 (GL Sciences Inc., Tokyo, Japan) connected to the module.

After 48 h of incubation, the gasbags were removed. Ten mL of gas was extracted from each gasbag using a gas-tight 10 mL 1010SL syringe (Hamilton, Bonaduz, Switzerland) and transferred into evacuated GC-vials (Labco Limited, Ceredigion, United Kingdom) for analysis of methane concentration. An aliquot of the rumen fluid was collected for VFA and Liquid Chromatography-Tandem Mass Spectrometry (LC–MS/MS) analyses. An aliquot of the buffered rumen fluid has also been collected prior incubation.

### Methane and VFA analyses using GC-TCD

Methane concentrations in gas samples were analysed using a Trace 1310 GC with a TCD detector and a TriPlus Headspace autosampler (Thermo Fisher Scientific, Waltham, MA, USA) as described by Jensen et al.^29^. VFA analyses were performed on the liquid flow-through collected during filtration by GC–MS as described by Olijhoek et al*.*^30^.

### Chemical composition of the standard feed

The MS used as standard feed had the following chemical composition (g/kg DM): organic matter (OM), 965; neutral detergent fiber (NDF), 329; starch, 351; crude protein, 77.7^31^.

### Liquid chromatography-tandem mass spectrometry (LC–MS/MS)

The concentrations of CAT, EPIC, QUE, RUT, GAL and SALA after 48 h of incubation were measured using a triple quadrupole mass spectrometer connected to a microLC according to the microLC-MS/MS method^32^ and adjusted to rumen fluid. The following standards were purchased from Sigma-Aldrich (Merck KGaA, Darmstadt, Germany), chemical purity (CP) and CAS number listed below: catechin ≥ 98% (18829-70-4), epicatechin ≥ 98% (490-46-0), quercetin ≥ 95% (117-39-5), rutin ≥ 94% (207671-50-9) and salicylic acid 99% (69-72-7) and labelled standards catechin-2,3,4-^13^C_3_ 99 atom % ^13^C (98% CP), salicylic acid-D_4_ certified reference material (78646-17-0) and enterolactone-2,3,5-^13^C3 (918502-72-4) from Toronto Research Chemicals (Toronto, ON, Canada). Two working solutions were prepared, one containing all non-labelled standards (ST mix) and another one containing the labelled standards (IS mix) in a working solvent of 5% ACN (v/v) and 1% FA (v/v) in pure water. The labelled standards were used as internal standards (IS). The standard curve was constructed to contain all the labelled and non-labelled compounds. The analyte/internal standard concentration ratio was plotted against the analyte/internal standard peak area ratio as a linear regression curve with 1/× weighting. The concentrations were calculated based on standard curve in Analyst software 1.7.1 from AB Sciex (Framingham, MA, USA). The chromatographic separation was performed on microLC 200 series from Eksigent/AB Sciex (Redwood City, CA, USA) coupled with a QTrap 5500 mass spectrometer from AB Sciex (Framingham, MA, USA). For chromatographic separation a Kinetex 1.7 µm Phenyl-Hexyl has been used with column oven temperature of 30 °C and autosampler racks of 20 °C. Mobile phases consisted of solvent A (1% FA (v/v) in pure water) and solvent B (0.1% FA (v/v) in ACN). The gradient started at 10% of solvent B for 0.5 min., followed by an increase in solvent B for 9 min. until 90% solvent B and was kept isocratic for 0.5 min. The columns equilibration time was 3 min with 10% of solvent B at the beginning of each run. The sample injection was 5 µL, and the flow of the system was 60 µL/min. The PCs were measured in MRM mode. The compound-dependent parameters were optimized manually for each compound by syringe infusion of pure standard and are shown in Supplementary Table S1. The dwell time was set to 15 ms, and the Entrance Potential (EP) was at − 10 eV. The ionization of compounds was performed with ESI in negative ionization mode, and the turbo V source of the instrument was optimized using Flow Injection Analyses (FIA). The source parameters were the following: curtain gas 30 psig, nitrogen gas 1 50 psig, nitrogen gas 2 40 psig, temperature 500 °C, ionization spray operated at − 4000 eV, and collision gas was set to High. Nitrogen was used as a source and collision gas. The data analysis was performed using Analyst software 1.7.1 from AB Sciex (Framingham, MA, USA). Prior LC–MS/MS analyses the rumen fluid samples were centrifuged at 4 °C for 10 min at 20,000×*g* and diluted ten times. The samples were diluted with 5% acetonitrile containing 1% formic acid and internal standards in pure water. The analyses of TAN and SALI were not available in our laboratory.

### Statistical analyses and calculations

The cumulative gas production (psi) data recorded during the 48 h of incubation were converted into volume (mL) of gas produced at standard temperature (0 °C) and pressure (1 bar) using the ideal gas law. TGP was blank corrected before the statistical analyses. The volume of methane (mL) produced were calculated multiplying their concentrations (%) in the collected gas with the TGP (mL).

The data for the various response parameters (TGP, methane, VFA) were statistically analysed by the Mixed procedure of SAS 9.4 (SAS Institute Inc., Cary, NC USA). The following model was used:

$$Y_{ijk} = \, \mu \, + \, T_{i} + \, R_{j} + \, B_{k} \left( R \right) + \, e_{ijk} ,$$

where *Y*_*ij*_ is the dependent response variable, *µ* is the overall mean, *T*_*i*_ is the fixed effect of sample type i.e. MS with or without addition of PSMs (*i* = 1 to 10; MS, MS-DMSO, CAT, EPIC, QUE, RUT, GAL, SALA, SALI, TAN), *R*_*j*_ is the random effect of experimental run (*j* = 1 and 2), *B*_*k*_(*R*) is the random effect of the bottle (*k* = 1–50) within the run, and *e*_*ijk*_ is the residual error. To evaluate the effect of the solvents (water or DMSO) used to dissolve the PSMs (additives), the two controls (MS and MS-DMSO) were compared using the Fisher’s least significant difference (LSD) test. Since there was no difference between MS and MS-DMSO for any variables tested, mean values of MS and MS-DMSO for each run were calculated and used as control in the subsequent statistical analysis.

Differences between additives were evaluated with Tukey adjustment. For all statistical analyses, significance was declared at *p* ≤ 0.05 and trend at 0.05 < *p* ≤ 0.10. Data in the tables are presented as least squares means and SEM.

Degradation of CAT, EPIC, QUE, RUT, GAL and SALA after 48 h of incubation using the concentrations measured by LC–MS/MS were calculated as follows:

$$Deg\% \, = \, 100 \, {-} \, (C_{end} /C_{start} \times 100),$$

where *Deg%* is the degradation (%) of the compound, *C*_*end*_ is the concentration of the compound after 48 h of incubation, and *C*_*start*_ is the concentration of a compound in the buffered rumen fluid solution added to bottles at the beginning of the incubation.

## Results

### Total gas and methane production

The results for TGP and methane production for each PSM added to the in vitro gas production system with inclusion dose of 12% (w/w DM of MS) are reported in Table 1. QUE, TAN and SALA induced significant reductions of TGP (mL/g DM and mL/g OM), when added to MS as compared to control, *p* = 0.001.

**Table 1:** Total gas production (TGP) and methane (CH_4_) production (mL per g of dry matter (DM) or organic matter (OM), and percentage (%) of TGP for plant secondary metabolites (PSMs) incubated for 48 h in an in vitro system simulating rumen fermentation. Each additive was incubated with maize silage, used as control feed at the inclusion dose of 12% (w/w DM).

PSM class	Additives	TGP (mL/g DM)	TGP (mL/g OM)	CH_4_ (mL/g DM)	CH_4_ (mL/g OM)	CH_4_ (% TGP)
–	Control	155^a,b^	159^a,b^	16.8^b^	17.3^b^	10.8^b^
PCs						
PA	GAL	150^a,b,c^	154^a,b,c^	15.7^b,c^	16.1^b,c^	10.4^b,c^
PA	SALA	126^d^	129^d^	11.1^c,d^	11.4^c,d^	8.73^b,c,d^
HT	TAN	129^c,d^	133^c,d^	10.1^d^	10.4^d^	7.86^c,d^
FAL	CAT	140^b,c,d^	144^b,c,d^	14.3^b,c,d^	14.6^b,c,d^	10.1^b,c^
FAL	EPIC	154^a,b^	158^a,b^	17.3^a,b^	17.8^a,b^	11.2^a,b^
FOL	QUE	130^c,d^	134^c,d^	9.47^d^	9.72^d^	7.25^d^
FOL	RUT	162^a^	166^a^	16.9^a,b^	17.4^a,b^	10.4^b,c^
Non-PC	SALI	162^a^	166^a^	21.4^a^	22.0^a^	13.1^a^
	SE	8.0	8.3	1.58	1.63	0.61
	*p*-value	< 0.001	< 0.001	< 0.001	< 0.001	< 0.001

*GAL* gallic acid, *SALA* salicylic acid, *TAN* tannic acid, *CAT* catechin, *EPIC* epicatechin, *QUE* quercetin, *RUT* rutin, *SALI* salicin, *PCs* Phenolic compounds, *PA* Phenolic acid, *HT* Hydrolysable tannin, *FAL* Flavanol, *FOL* Flavonol, *Non-PC* Non-phenolic compound, *SE* Standard Error. ^abcd^Statistically different values compared to the control (*p* ≤ 0.05) after Tukey adjustment, with comparison performed within the column.

Methane production (mL/g DM and mL/g OM) was significantly reduced by 43% with addition of QUE (but not the other flavonoids CAT, EPIC or RUT), 39% by TAN (but not its subunit GAL) and 34% by SALA compared to control (MS without PSM addition), *p* = 0.001 (Table 1). This was caused by a reduction in methane concentration in the gas produced during fermentation. QUE, TAN and SALA belong to three different classes of compounds, flavonols (flavonoids), hydrolysable tannins (non-flavonoids) and phenolic acids (non-flavonoids), respectively (Fig. 1).

Oppositely to QUE, TAN and SALA which reduced methane significantly, SALI increased methane production by 21% compared to control, *p* = 0.001 (Table 1). Methane production was more than twice as high with SALI added to MS as compared to methane production after addition of QUE, TAN and SALA with other PSMs falling in between.

**Figure 2:**
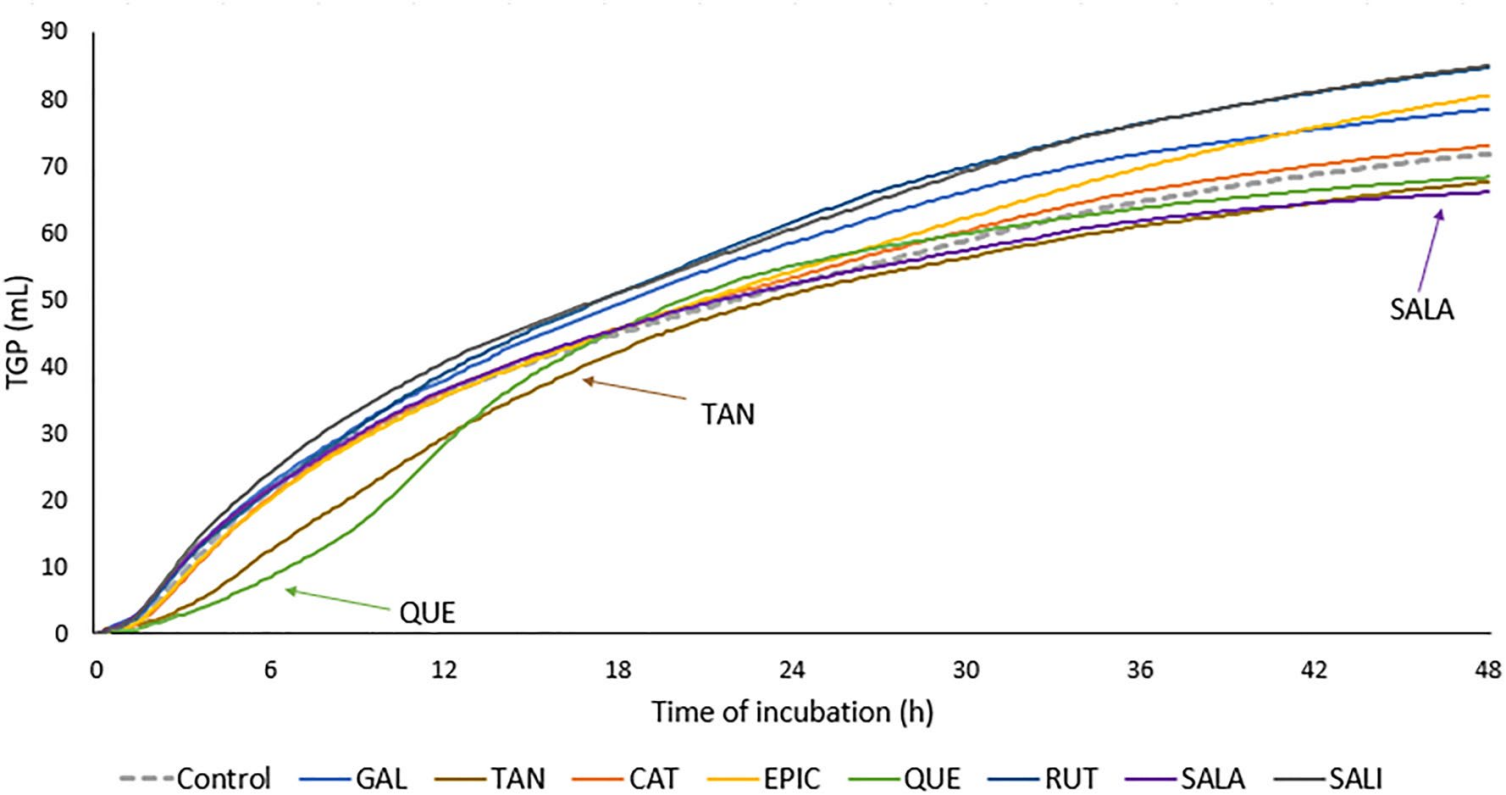
Accumulated total gas production (TGP) over 48 h of incubation in buffered rumen fluid of maize silage (MS) without (control) or with addition of plant secondary metabolites (PSMs) representing different classes of phenolic compounds (PCs) and a non-PC. Phenolic acids: gallic acid (GAL) and salicylic acid (SALA); hydrolysable tannin: tannic acid (TAN); flavanols: catechin (CAT) and epicatechin (EPIC); flavonols: quercetin (QUE) and rutin (RUT); and non-PC: salicin (SALI). Classification of PSMs is shown in Fig. 1.

The development in accumulated TGP during the 48 h of incubation is shown in Fig. 2. We have observed clear differences in TGP curves development among the tested PSMs. TGP developed more slowly during the first 15–18 h of fermentation when QUE (but not the other flavonoids CAT, EPIC or RUT) and TAN (but not its subunit GAL) had been added to MS. In contrast to QUE and TAN accumulated TGP developed slower by the end of incubation when SALA was incubated with MS.

### Rumen fermentation characteristics

The impact of addition of each PSM to MS at an inclusion dose of 12% (w/w DM of MS) on production of VFA during fermentation are reported in Table 2. Lowered methane formation by QUE, TAN and SALA was not associated with changes in total VFA production compared to control but was associated with changes in composition of the individual VFA, *p* = 0.001 (Table 2). QUE significantly increased the proportion of acetic acid but reduced the proportions of butyric and iso-butyric acids as well as total other VFA. In contrast, TAN reduced the proportion of acetic acid but increased the proportion of propionic acid, *p* = 0.001 (Table 2). Similar to QUE, TAN reduced the proportions of butyric and iso-butyric acids as well as total other VFA *p* = 0.001 (Table 2). SALA did not induce significant changes on total VFA production nor the individual VFA concentrations, except for the concentration of total other VFA.

**Table 2:** Total Volatile fatty acid (VFA) concentrations for plant secondary metabolites (PSM) in the in vitro system simulation rumen fermentation during 48 h of incubation. Acetic, propionic and butyric acids were further calculated as percentage (%) of total VFA. Valeric, Isovaleric and caproic acids were further calculated as percentage (%) of total other VFA. Each additive was incubated with maize silage, used as control feed at the inclusion dose of 12% (w/w DM).

PSM class	Additives	Total VFA (mmol/L)	Acetic acid (%)	Propionic acid (%)	Iso-butyric acid (%)	Butyric acid (%)	Total other VFA (mmol/L)	Total other VFA (%)
–	Control	68.4^d,e^	69.2^b,c^	14.9^c,d^	1.15^a^	11.4^a^	2.31^a,b^	3.37^a^
PCs								
PA	GAL	72.0^b,c^	70.7^a,b^	14.5^c,d^	1.03^c^	10.7^b,c^	2.21^b,c^	3.06^b,c^
PA	SALA	66.4^e^	70.2^b,c^	14.3^c,d^	1.06^b,c^	11.2^a,b^	2.10^c^	3.17^a,b,c^
HT	TAN	66.0^e^	67.2^d^	17.9^a^	1.06^b,c^	10.7^b,c^	2.06^c^	3.08^b,c^
FAL	CAT	73.1^a,b^	72.4^a^	14.1^c,d^	1.01^c^	9.64^d^	2.09^c^	2.85^c^
FAL	EPIC	75.1^a^	72.3^a^	14.0^c,d^	1.03^c^	9.75^d^	2.19^b,c^	2.91^c^
FOL	QUE	69.5^c,d^	72.2^a^	13.9^d^	0.817^d^	10.8^b,c^	1.60^d^	2.29^d^
FOL	RUT	75.3^a^	69.7^b,c^	16.3^b^	0.968^c^	10.1^c,d^	2.16^b,c^	2.87^c^
Non-PC	SALI	72.6^a,b^	68.5^c,d^	15.6^b,c^	1.13^a,b^	11.4^a,b^	2.47^a^	3.39^a,b^
	SE	2.05	0.88	0.56	0.073	0.87	0.288	0.329
	*p*-value	< 0.001	< 0.001	< 0.001	< 0.001	< 0.001	< 0.001	< 0.001

*Total VFA* sum of acetic, propionic, butyric, iso-butyric, valeric, iso-valeric and caproic acids, *Total other VFA* sum of valeric, isovaleric and caproic acids, *GAL* gallic acid, *SALA* salicylic acid, *TAN* tannic acid, *CAT* catechin, *EPIC* epicatechin, *QUE* quercetin, *RUT* rutin, *SALI* salicin, *PCs* Phenolic compounds, *PA* Phenolic acid, *HT* Hydrolysable tannin, *FAL* Flavanol, *FOL* Flavonol, *Non-PC* Non-phenolic compound, *SE* Standard Error. ^abcde^Statistically different values compared to the control (*p* ≤ 0.05) after Tukey adjustment, with comparison performed within the column.

The highest total VFA concentration by the end of fermentation was measured for the flavanols, CAT (73.1 mmol/L) and EPIC (75.1 mmol/L), the flavonol, RUT (75.3 mmol/L), and the non-PC, SALI (72.6 mmol/L), Table 2. This was significantly higher, *p* = 0.001, than the lowest measured total VFA concentrations of 66 mmol/L when TAN had been added to MS.

There were significant systematic changes in the composition of VFA induced by PSMs in two cases. Low total VFA concentrations with addition of TAN (66 mmol/L) was associated with the lowest observed proportion in VFA of acetic acid, 67.2%, with a shift towards highest proportions of propionic acid 17.9%. Furthermore, the addition of CAT and EPIC to MS gave rise to high total VFA concentrations (73.1 and 75.1 mmol/L, respectively) including the highest observed proportion of acetic acid, 72.4 and 72.3%, respectively, but the lowest observed proportion of butyric acid, 9.64 and 9.75%, respectively.

Overall, the majority of tested PSMs reduced the proportions of butyric and iso-butyric acids significantly, with exception of SALA and SALI. TAN and RUT were the only PSMs which increased the proportion of propionic acid.

### Degradability of PSMs

High degradability of PSMs was observed after 48 h of incubation in buffered rumen fluid in vitro (Table 3). The degradation was 100% for the flavonols, QUE and RUT, whereas it was slightly lower (94–99%) for flavanols, CAT and EPIC. Phenolic acids had lower degradability compared to flavonoids, with SALA having the lowest degradability among the PCs, though with considerable difference between the two in vitro incubation runs varying between 14 and 32%. The degradability of GAL was around 90%.

**Table 3:** Mean concentration and degradation of plant secondary metabolites (PSMs) after 48 h of incubation in buffered rumen fluid in vitro, n = 3 of 2 runs (r).

PSM class PCs	Additive	Concentration (µg/mL) r = 1	Degradation (%) r = 1	Concentration (µg/mL) r = 2	Degradation (%) r = 2
	Control	–	–	–	–
PA	GAL	73	90	56	92
PA	SALA	600	14	479	32
FAL	CAT	39	94	32	95
FAL	EPIC	22	97	10	99
FOL	QUE	0.4	~ 100	0.2	~ 100
FOL	RUT	< LLOQ*	~ 100	< LLOQ*	~ 100

*GAL* gallic acid, *SALA* salicylic acid, *CAT* catechin, *EPIC* epicatechin, *QUE* quercitin, *RUT* rutin; *Lower Limit of Quantification (LLOQ); *PCs* Phenolic compounds, *PA* Phenolic acid, *FAL* Flavanol, *FOL* Flavonol.

## Discussion

Although the anti-methanogenic action of PSMs is not well studied, there is general agreement in the literature that PCs such as tannins, flavonoids and phenolic acids act as anti-methanogenic agents either through direct action on methanogens or through indirect action on protozoa to which some methanogens are associated and which are important for their function^7, 9, 18^.

### Flavanols and flavonols

Lipophilicity is an important chemical characteristic of PCs, which can enhance their anti-microbial activity by favouring their interaction with the bacterial cell membrane^33^. This interaction results in inhibition of the bacterial cytoplasmic membrane function, bacterial cell wall synthesis or inhibition of nucleic acid synthesis^18, 34^. In this study, QUE exhibited strong anti-methanogenic activity, whereas no similar activity was observed for the structurally similar CAT, EPIC and to some extent RUT. Although QUE, CAT and EPIC are flavonoids with a similar number of hydroxyl groups and approximately similar MW, QUE is more lipophilic compared to CAT and EPIC, which is related to structural differences of the molecules. Based on their molecular structures QUE is classified as a flavonol, whereas CAT and EPIC belong to the class of flavanols. The molecular structure of QUE is characterized by the double bond and carbonyl group in the C ring of the molecule. The more water-soluble CAT and EPIC lack this double bond and carbonyl group in the C ring^20^, and it may therefore be suggested as an important structural difference between these compounds which could play a role in the mode of action against methane formation. According to the study of Oskoueian et al.^18^, QUE significantly suppressed the population of total protozoa and total methanogens, whereas CAT significantly reduced the populations of almost all rumen microorganisms. This again indicates differences in the modes of action between QUE and CAT with regard to anti-microbial activity. In agreement with our study QUE has previously been reported as a potent anti-methanogenic compound in vitro by both Sinz et al.^35^ and Oskoueian et al.^18^. Sinz et al.^35^ reported anti-methanogenic effects of EPIC in contrast to our study, but also observed that this was dose dependent. Thus, the methane inhibiting activity was only observed at doses from 5 to 50 mg/g DM of basal diet and not at 0.5 mg/g DM^35^. Becker et al.^36^ reported that CAT was a potent anti-methanogenic compound, opposite to the findings in our study as well as the studies by Sinz et al.^35^ and Oskoueian et al.^18^. Overall, there is good agreement in the literature that QUE acts as an anti-methanogenic agent in vitro, but not with regard to CAT and EPIC. Further studies are warranted to understand the structure–function relationships underlying differences in influence of QUE compared to CAT and EPIC on methane formation.

Both CAT and EPIC, significantly increased the total VFA production compared to control, presumably as a result of fermentation of these compounds in the rumen fluid, and hence they directly or indirectly influenced microbial fermentation patterns as indicated by an increasing proportion in VFA of acetic acid and decreased proportions of butyric and iso-butyric acids as well as total other minor VFAs. QUE did not change the production of total VFA, but the composition of VFA was similar to that observed with CAT and EPIC. It can be assumed that due to high degradation of QUE, CAT and EPIC (≥ 94%) by rumen microorganisms, these compounds may themselves have been utilized by rumen microbes and contributed to VFA production in a distinct manner. Contrary to our results, other work did not find a significant impact on total VFA production, when CAT^18, 36^ or EPIC^35^ were incubated with feeds in vitro. The differences in these findings may relate to the dose of inclusion. We used a higher dose of 12% (w/w DM of MS) of CAT and EPIC, which may have triggered measurable increases in VFA production in our study, compared to the maximum dose of 4.5–5% (w/w of DM of dry guinea grass and concentrate in the ratio of 60:40) in the studies of Oskoueian et al*.*^18^ and Sinz et al.^35^ (w/w DM of ryegrass hay). Overall, flavonoids QUE, CAT and EPIC tended to increase acetate production at the expense of butyrate.

Although, both QUE and RUT belong to the same class of compounds known as flavonols, they have exhibited significantly different effects on methane and VFA production. QUE was shown to have an anti-methanogenic potential, whereas RUT did not reduce methane production compared to the control. RUT on the other hand, significantly increased total VFA production compared to the control, whereas QUE did not. The difference in the effects of QUE and RUT on rumen fermentation may be related to the difference in the molecular structures of the two compounds. RUT contains a sugar moiety^18^, whereas QUE is an aglycon. The possible explanations for the differential effects of RUT compared to QUE could be related to either: (a) interference with anti-methanogenic properties of the 3-ring backbone due to addition in RUT of the rather large sugar moiety^18^, or (b) degradation of the β-glycosidic bond in RUT by bacterial β-glucuronidases, hence liberating and delivering sugar as a substrate for microbial fermentation^37^. We have previously shown that flavonoid glycoside could influence microbial fermentation and increase the production of butyric acid when extracted and fractionated from hemp *Cannabis sativa* Futura 75^29^. The increased total VFA production by RUT can thereby be linked to its degradation (~ 100%) and the subsequent availability of sugars used by rumen microorganisms for fermentation and hydrogen for production of propionic acid. In the in vitro study by Oskoueian et al*.*^18^, the effects of QUE, RUT and other PSMs on rumen fermentation were compared at 4.5% (w/w DM) addition to feed, and they found that both RUT and QUE significantly increased TGP, but decreased methane without affecting VFA production. In our study, QUE significantly reduced methane without affecting the total VFA production, but no similar properties could be assigned to RUT. The differences in the results could be assigned to the lower inclusion dose 4.5% (w/w DM) used in the study of Oskoueian et al.^18^ compared to our study 12% (w/w DM)*.* Therefore, further studies of the influence of glucosidation are also warranted.

### Non-PC

SALI was another compound, which induced higher total VFA production compared to control associated with an actual increase in methane formation. SALI is a non-PC with a molecular structure in which a phenolic hydrogen is substituted with a sugar moiety. Similar to RUT, release of the sugar moiety by rumen bacterial enzymes could potentially contribute to increased fermentation and hence methane formation. In monogastric animals and humans salicin is degraded by intestinal enzymes and bacteria to saligenin and glucose. Saligenin is further oxidized in the blood and liver to SALA^38^. A low concentration of SALA (LC–MS data not shown) was detected in the fermented rumen fluid after incubation of the standard feed with SALI, and this indicated that rumen microbes can convert SALI to SALA. To our knowledge, there are no other in vitro studies on the anti-methanogenic potential of SALI. Our study showed that SALI increased methane and total VFA production by 21% and 6% respectively, indicating its high fermentability when incubated in the buffered rumen fluid at a dose of 12% (w/w DM of MS) for 48 h.

### Phenolic acids

Both SALA and GAL are low MW compounds, weak acids containing a carboxyl group, but they differ in their lipophilicity. SALA is more lipophilic with only one hydroxyl group compared to GAL, which contains three hydroxyl groups. Higher lipophilicity has been shown to be an important chemical characteristic determining the anti-microbial activity of PCs^33^ including phenolic acids^39^. The anti-microbial activity of phenolic acids has been demonstrated previously. The mechanism of action of phenolic acids has been explained by the diffusion across the microbial membrane, resulting in acidification of the cytoplasm and cell death^40, 41^. Consequently, acidity and lipophilicity has been suggested to determine the solubility of phenolic acids in bacterial membranes and thus their anti-microbial activity^40, 41^. SALA but not GAL reduced methane formation significantly and lipophilicity may have been an important chemical characteristic for this anti-methanogenic effect, whereas the acidity of the carboxyl group did not appear to play a role in this action. On the other hand, GAL significantly increased total VFA production, whereas SALA did not affect VFA production or composition. This can be related to differences in rumen degradability, which for SALA was low (14 and 32%) resulting also in a low accumulated TGP by the end of fermentation, Fig. 2. Our study is the first to show an anti-methanogenic potential of SALA.

### Hydrolysable tannin

Interestingly, the high MW TAN was able to significantly reduce enteric methane formation, whereas its low MW monomeric subunit GAL was not. The importance of MW of tannins was previously demonstrated in vitro by Tavendale et al.^42^ and Saminathan et al.^43^. These studies showed that lower MW fractions of condensed tannins were less effective in reducing the total population of methanogens than high MW fractions of condensed tannins. Generally, it is assumed that the higher MW the greater the general binding ability of tannins to proteins and carbohydrates^23^. When evaluating the accumulated gas production curves from our study, it was clear that the impact of TAN on gas production was highest in the early phase of fermentation as also seen for QUE, Fig. 2. It remains speculation whether the methane inhibiting effect would have followed a similar pattern. TAN is a polymer which is quickly degraded by rumen microbes into its individual subunits^35, 44, 45^, thus explaining why the impact on gas production disappeared over time, as the non-functional (against methane) GAL units became liberated. In the fermented fluid sampled post-fermentation, a low concentration of GAL (LC–MS/MS data not shown) was measured in bottles incubated with TAN in our study, indicating the degradation of TAN to GALA. The relationship between degree of polymerization of tannins and their monomeric subunits to methane inhibiting properties requires further studying.

It has been shown previously that hydrolysable tannins are able to alter ruminal fermentation^46^. TAN did not change the total VFA production, however it is the only PSM that increased the proportion of propionic acid while it decreased the proportions acetic acid, butyric and iso-butyric acids and total other minor VFAs. The increased proportion of propionic acid can be related to the methane inhibiting effect of TAN, which would cause increased availability of hydrogen in the rumen liquid to become available for the hydrogen consuming pathway leading to production of propionic acid^47, 48^.

## Conclusion

In this in vitro study, we have observed clear anti-methanogenic effects when QUE, TAN and SALA were co-fermented in rumen inoculum with MS at a dose of 12% (w/w DM) and without any associated effects on the production of total VFA. None of the other tested PSMs in this study showed significant anti-methanogenic effects, while they did affect fermentation patterns of the individual VFA. Further, we observed a significant association between chemical characteristics and molecular structures of PSMs on methane and VFA formations. Lipophilicity was the main chemical characteristic that could be related to anti-methanogenic activity of PSMs. The main molecular structure related to an anti-methanogenic activity was the presence of a double bond and carbonyl group in the molecule of QUE. The presence of sugar moieties would provide substrate to fermentation and resulted in increased formation of both VFA and methane. Presence of carboxyl groups on the phenolic acids did not seem to have any effect on methane formation. However, further studies are warranted to systematically validate relationships between different physicochemical properties of PSMs and methane reduction and to investigate the relationship between doses of inclusion and different types of substrates for incubation. This will allow us to understand the mode of action of PSMs on methane reduction and their possible future application for in vivo studies.

### Supplementary Information

Supplementary Information.

## Data Availability

The data presented in this study are available on request from the corresponding author. The data are not publicly available due to the security policy of Aarhus University.
