# Supplementary material for: Methane reduction by quercetin, tannic and salicylic acids: influence of molecular structures on methane formation and fermentation in vitro
Source: Sci Rep. 2023 Sep 25;13:16023. doi: 10.1038/s41598-023-43041-w (PMC10519955; doi:10.1038/s41598-023-43041-w)
Supplement: Supplementary file 1 — Supplementary Information. [file 41598_2023_43041_MOESM1_ESM.pdf]

Methane reduction by quercetin, tannic and salicylic acids: influence of molecular structures on methane formation and fermentation in vitro

Natalja P. Nørskov [1*], Marco Battelli [2], Mihai V. Curtasu [1], Dana W. Olijhoek [1], Élisabeth Chassé [1] and Mette Olaf Nielsen [1]

[1] Department of Animal and Veterinary Sciences, Aarhus University, Blichers Allé 20, 8830 Tjele, Denmark; mihai.curtasu@anivet.au.dk (M.C.); dana.olijhoek@anivet.au.dk (D.W.O.); elisabeth.chasse@anivet.au.dk (E.C.); mon@anivet.au.dk (M.O.N.); natalja.norskov@anivet.au.dk (N.P.N.)

[2] Department of Agricultural and Environmental Sciences - Production, Landscape, Agroenergy, Università degli Studi di Milano, via Celoria 2, 20133 Milan, Italy; marco.battelli@unimi.it (M.B.)

* Correspondence: natalja.norskov@anivet.au.dk; Tel.: +45-30563085

SUPPLEMENTARY MATERIALS

Table S1. Compound-dependent parameters optimized by syringe infusion of pure standards. Declustering Potential (DP), Collision Energy (CE), and Cell Exit Potential (CEP).

Compounds	Q1 mass (m/z)	DP (V)	Q3 mass (m/z)[a]	CE (eV)	CXP (V)
Catechin/ Epicatechin	289.2	-125	**108.8**/123.0	-29/-39	-8/-15
Catechin-2,3,4-$^{13}C_3$	292.0	-135	**108.8**	-30	-8
Rutin	609.2	-203	**300.0**/270.9	-50/-69	-17/-22
Quercetin	300.9	-133	**150.9**/106.9	-27/-38	-14/-11
Enterolactone-2,3,5-$^{13}C_3$	300.0	-128	**191.9**	-30	-14
Enterodiol-1,2,4-$^{13}C_3$	304.1	-140	**255.1**	-32	-17
Salicylic acid	136.9	-36	**93.0**	-20	-7

Salicylic acid-D_4	140.9	-35	**97.0**	-21	-10
Gallic acid	168.9	-40	**124.9**	-20	-18

[a] quantifier ions are given in bold.

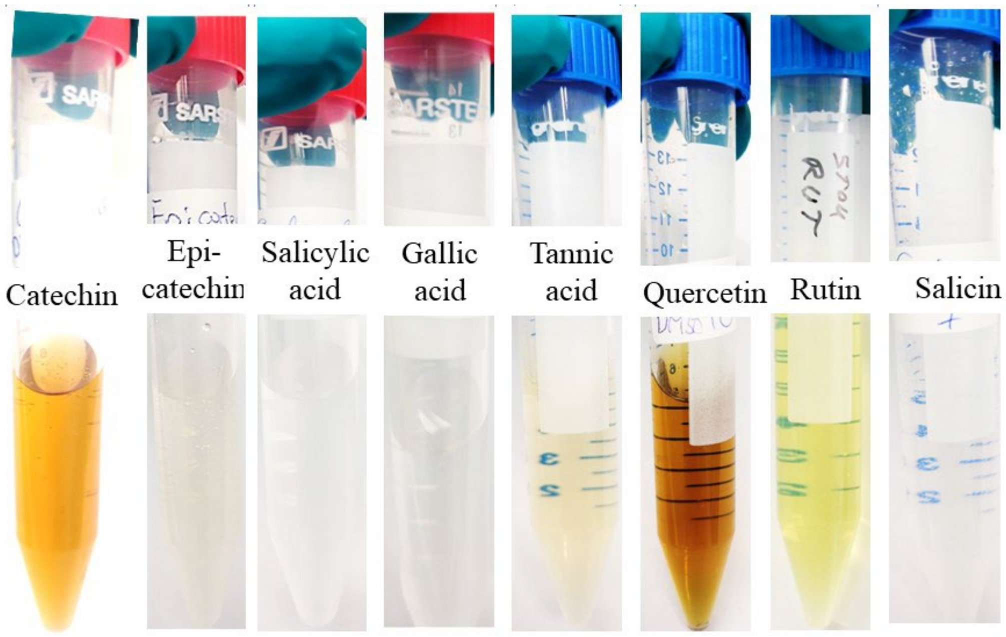

Figure S1. Plant Secondary Metabolites (PSMs) dissolved in either water or dimethyl sulfoxide (DMSO), 2 mL of each PSM was added to in vitro fermentation system.

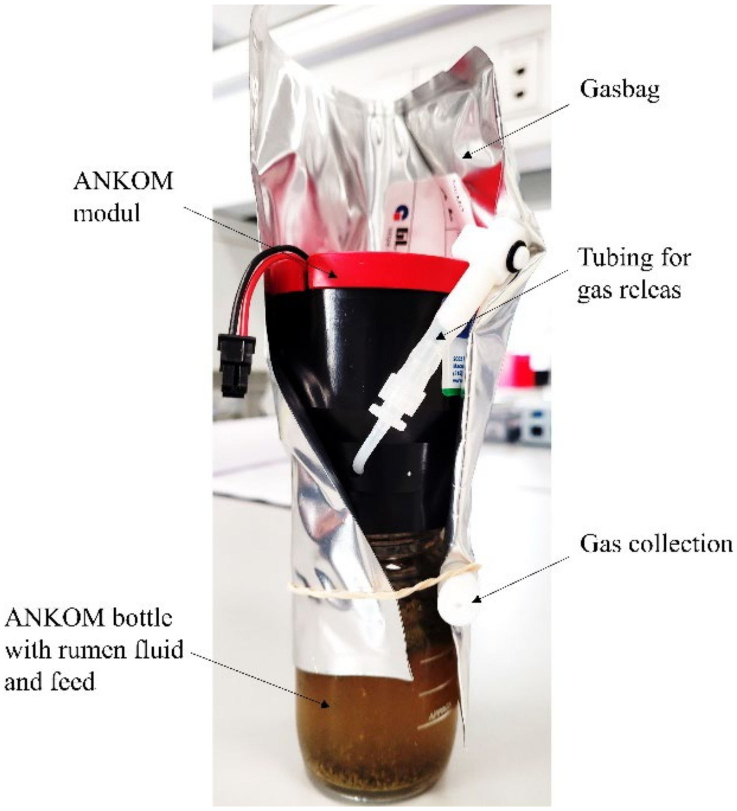

Figure S2. In vitro system simulating rumen fermentation (ANKOM®).
